# A Case Report of Sjögren’s Syndrome Presenting With Autoimmune Hepatitis

**DOI:** 10.7759/cureus.23464

**Published:** 2022-03-24

**Authors:** Rachael Caretti, Caroline Wojtas, Mojdeh Baniasadi, Liana Milis, Randy Scott

**Affiliations:** 1 Medicine, Lake Erie College of Osteopathic Medicine (LECOM), Bradenton, USA; 2 Family Medicine, Lake Erie College of Osteopathic Medicine (LECOM), Bradenton, USA

**Keywords:** anti-ro/ssa, abnormal liver function test, autoimmune syndromes, autoimmune hepatitis, sjögren’s syndrome

## Abstract

Sjögren’s syndrome (SS) is the result of an abnormal immune response in the body that leads to the destruction of exocrine glands, primarily the ocular and salivary glands, resulting in xerophthalmia and xerostomia. We describe a case of a 55-year-old female who presented to the clinic with abnormal liver function tests, identified on routine blood work. After an extensive workup, the patient was diagnosed with autoimmune hepatitis (AIH) and SS. AIH occurs in a small number of patients with primary SS, and there is little research available on the overlap of these two autoimmune disorders. In this report, we will present some of the challenges in diagnosing AIH and identifying SS as the underlying diagnosis. Because of the ambiguous clinical picture, diagnosis of AIH can be difficult, especially in the setting of concomitant autoimmune disorders.

## Introduction

Pathophysiology: Sjögren’s syndrome

Sjögren’s syndrome (SS) is an autoimmune disease that affects approximately 0.5% of the population. With women disproportionately impacted, the female to male ratio is approximately 9:1 [1]. Of this portion of the population, only a small number will develop autoimmune hepatitis (AIH), in addition to SS, which is estimated to be 1% [2]. Because of the rarity of having concomitant diseases, diagnosis may be overlooked or delayed.

SS leads to inflammation of exocrine glands through an abnormal immune response of B and T cells to autoantigens, for example, anti-Ro/Sjögren's-syndrome-related antigen A (SSA) or anti-La/Sjögren's-syndrome-related antigen B (SSB), ultimately leading to the destruction of the glands [3]. It is theorized that there may also be an X-linked chromosomal component to the disease because of the high preponderance of females affected. These factors may influence apoptosis, which leads to the release of chemokines, as well as upregulating adhesive molecules that direct lymphoid migration to glandular tissues [1]. Others have hypothesized that there may be a link between amyloidosis and SS in which permeability of plasma cells is affected by amyloid deposition [4]. The resulting destruction of glandular tissue is primarily in ocular and salivary glands, which presents as xerophthalmia and xerostomia [3].

There is bimodal age distribution for peak ages of onset in patients with SS: the first range from 20 to 30 years of age and the second in the mid-50s. Patients may also present with extraglandular manifestations, such as arthralgias and myalgias. SS also predisposes patients to complications, such as dental caries, gastroesophageal reflux disease (GERD), pneumonitis, interstitial nephritis, and lymphoma [1].

SS can be initially treated with corticosteroids but they should be used with caution due to the increased risk of dental caries and candidiasis [1]. Medications that are typically used to taper patients off corticosteroids include hydroxychloroquine, azathioprine, or methotrexate. It is important to note that no immunomodulatory drug has been proven to be efficacious in treating SS, but they are nonetheless generally used for treatment due to open-label studies showing decreased symptoms with hydroxychloroquine [5].

Pathophysiology: autoimmune hepatitis

AIH can present as fulminant hepatitis, non-specific symptoms, or be asymptomatic [6]. Because AIH has such a wide variety of clinical presentations, diagnosis can be difficult but is important because untreated AIH has five-year mortality above 50% [7]. With any patient presenting with elevated liver enzymes, or with cirrhosis, AIH should be included as a differential [8]. AIH is classified as either type 1 or type 2, based on the antibodies involved. For this review, type 1 AIH will be discussed; it is more common than type 2 and likely the type of AIH in the above-described case. Type 1 AIH is associated with the following circulating antibodies: antinuclear antibodies (ANA), smooth muscle antibodies, anti-actin antibodies, atypical perinuclear antineutrophilic cytoplasmic antibodies (pANCA), and autoantibodies against the soluble liver antigen and liver-pancreas antigen (SLA/LP) [9]. With growing evidence of a genetic component, there is also a theory that molecular mimicry and an imbalance within effector and regulatory immunity may play a role in disease pathology [6].

There is a wide spectrum of presentations of AIH and patients may present with symptoms of varying severity including fatigue, lethargy, malaise, weight loss, nausea, abdominal pain, and pruritus [9]. If left untreated, AIH has five-year mortality that is greater than 50%, making its diagnosis important yet difficult because there is no one specific test that can be diagnostic for all patients [7]. The risk of primary hepatocellular carcinoma is increased in patients with AIH because chronic hepatitis leads to cirrhosis, which ultimately results in carcinoma [9].

Because of the wide variety of clinical presentations, the International Autoimmune Hepatitis Group set forth the simplified diagnostic criteria in 2008 to aid in diagnosis [10]. Diagnostic criteria are based on autoantibodies, IgG levels, liver histology, and the absence of viral hepatitis. A score greater than or equal to seven yields a diagnosis of AIH and a score greater than or equal to six is probable AIH. In clinical practice, if there is a concern for drug-induced hepatitis vs. AIH, a trial of immunosuppressive therapy may be used. When the immunosuppressive therapy is stopped, patients with AIH will relapse whereas patients with drug-induced hepatitis will not relapse as long as the offending medication is not restarted [8].

The current guidelines published by the American Association for the Study of Liver Disease recommend that patients with AIH are initially treated with either a monotherapy of prednisone or dual therapy of prednisone with azathioprine. Once remission is achieved, azathioprine alone is used for maintenance therapy [8]. Patients who have remained in remission after a minimum of three years of treatment may be able to stop immunosuppressive therapy, but it is recommended that follow-up biopsies are performed prior to medication cessation [8].

## Case presentation

In March 2018, a 55-year-old Caucasian female first presented to the office to establish primary care. The physical exam was unremarkable. She was obese, with a BMI of 32.44. A baseline complete blood count (CBC), lipid panel, and comprehensive metabolic panel (CMP) were drawn. At the follow-up appointment to go over her lab results, the patient was diagnosed with hyperlipidemia. The patient’s aspartate aminotransferase (AST) and alanine aminotransferase (ALT) were within normal limits at that time, and she was started on 40 mg of atorvastatin.

In July 2018, the patient was seen again and repeat labs were drawn to monitor her lipids, ALT, and AST. At that time, the patient’s low-density lipoprotein (LDL) had decreased from 147 to 74 mg/dl. Her ALT was mildly elevated at 35, and her AST was within normal limits at 26. The decision was made to keep her on the current therapy and repeat her labs in a few months.

In December 2018, at the age of 56 years, the patient was seen again at the office. Her LDL had increased to 85, but she admitted to missing doses of the atorvastatin. Her ALT remained stable at 35, and her AST was within normal limits at 29.

The patient was not seen again until September 2019, now aged 57, for her annual visit and lab follow-up. At this visit, her AST had increased to 761 and her ALT to 1703. A viral hepatitis panel, repeat CMP, and right upper quadrant ultrasound were ordered. In early October 2019, the patient returned for a follow-up appointment. The viral hepatitis panel was negative and did not show prior vaccination against hepatitis B. Her AST had decreased from 761 to 281, and her ALT from 1703 to 868. The right upper quadrant ultrasound showed no masses. She was not taken off atorvastatin. At the end of October 2019, the patient had an additional lab follow-up, at which point her AST had increased to 330, and her ALT decreased to 643. She continued to be asymptomatic at this time.

In December 2019, there was a follow-up with another set of labs; her AST continued to drop to 171, and her ALT was 321. She was seen again in January of 2020 for a lab follow-up where she complained of hair loss. Thyroid labs were ordered. The patient’s liver enzymes began to trend upward again (AST = 302 and ALT = 561). Ordering an abdominal CT scan was discussed with the patient, but she declined and preferred to follow up with labs.

In March 2020, the patient was seen again. Her AST at this time was 409, and her ALT was 699. The decision to stop atorvastatin therapy was made and the patient was agreeable to an abdominal CT scan. The patient’s thyroid panel came back within normal limits. The patient was called in April 2020 and informed that the results of the abdominal CT scan were normal and that she should follow up with her scheduled lab appointment.

The patient was seen at the end of April 2020 and complained of two episodes of epistaxis in the week prior to her appointment. Her AST and ALT continued to increase to 443 and 699, respectively. At this time, the following additional labs were ordered: cytomegalovirus (CMV), Epstein-Barr virus (EBV), ANA with reflex, rheumatoid factor (RF), ceruloplasmin, IgM, erythrocyte sedimentation rate (ESR), and C-reactive protein (CRP). The possibility of a hepatology referral due to the negative workup was also discussed with the patient.

In May 2020, the patient was seen and complained of another episode of epistaxis that lasted 30 minutes. Upon further questioning, she admitted she has had fatigue, dry eyes, dry mouth, dry skin, and joint stiffness. It was also noted on examination that she had mild parotid and submandibular swelling, as well as conjunctival injection. The patient’s prior labs were negative, except for a positive ANA and an anti-SSA > 8. The patient was referred to rheumatology for further treatment and management of SS. Since the patient’s liver function tests (LFTs) were unchanged, despite discontinuing the atorvastatin, she was restarted on 40 mg due to the increase in her LDL cholesterol.

In June 2020, the patient was referred to rheumatology, where they agreed with the diagnosis of SS. They recommended her to follow up with ophthalmology and hepatology. The rheumatologist recommended delaying treatment of SS until the patient was evaluated by hepatology for the persistent elevation of LFTs. Later that month, she was evaluated by gastroenterology. Gastroenterology ordered labs to evaluate LFTs, as well as for EBV and hepatitis C RNA.

In July 2020, the patient followed up again with gastroenterology, at which point her EBV and hepatitis C were negative and her LFTs remained elevated. At this point, gastroenterology wanted to rule out hereditary hemochromatosis and AIH. Anti-smooth muscle antibody, an iron panel, and LFTs were ordered.

In September 2020, the patient followed up with gastroenterology; at this time, her ALT and AST decreased to 340 and 243. The physician discussed ordering an ANA and anti-smooth muscle antibody again since they were not completed after the last visit. A liver biopsy was recommended but the patient declined.

In September of 2020, the patient, now 58 years old, followed up for her annual wellness visit. At this time, the patient’s AST remained elevated at 310, and her ALT elevated at 423. The decision was made not to start her on methotrexate for SS until a further workup for her elevated LFTs was completed.

Following a liver biopsy, the patient followed up again with gastroenterology in October 2020. They ruled out hepatitis A, B, and C, as well as alcoholic hepatitis, primary biliary cirrhosis, and hereditary and acquired hemochromatosis. The liver biopsy pathology report showed the following: chronic inflammatory infiltrates and predominantly lymphocytes and plasma cells with no significant fat. There was some periportal and pericellular fibrosis on trichrome. Features were felt to be nonspecific and could include AIH, possibly with overlapping primary biliary cirrhosis, primary sclerosing cirrhosis, drug-induced liver injury, or infection. No specific autoimmune etiology was identified, but given her history of autoimmune disease, she likely had AIH, so treatment with prednisone 40 mg was started. Order for a CBC and CMP were written for labs to be drawn after starting the medication.

In November 2020, the patient followed up with gastroenterology and was diagnosed with probable AIH; diagnostic criteria are shown in Table 1. At this time, the AST and ALT decreased to 85 and 170, respectively. At that point, she was tapered off the prednisone and advised LFTs would be rechecked at her next appointment.

**Table 1:** Diagnostic criteria for autoimmune hepatitis. A score greater than or equal to 6 is probable AIH. A score greater than or equal to 7 is definite AIH [8]. AIH: autoimmune hepatitis; ANA: antinuclear antibodies; ASMA: anti-smooth muscle antibody; LKM = liver kidney microsomal antibody; SLA/LP: soluble liver antigen and liver-pancreas antigen.

		Points
Autoantibodies	ANA or ASMA or LKM > 1:80	2
	ANA or ASMA or LKM > 1:40	0
	SLA/LP positive (>20 units)	1
IgG (or gamma-globulins)	>1.10 times normal limit	2
	Upper normal limit	1
Liver histology	Typical for autoimmune hepatitis	2
	Compatible with autoimmune hepatitis	1
	Atypical for autoimmune	0
Absence of viral hepatitis	Yes	2
	No	0

At the beginning of January 2021, the patient followed up with primary care. At that time, her AST was within normal limits, and her ALT was mildly elevated at 47. It was recommended that she continued to see rheumatology and gastroenterology for further management of SS and AIH. At her next gastroenterology follow-up appointment later in January, she finished her prednisone taper, and her repeat AST and ALT results were 50 and 47, respectively. The decision was made to withhold any additional pharmacologic therapy and to repeat her LFTs in six weeks; the results of the lab work would guide the decision of whether to restart prednisone with azathioprine or to withhold pharmacologic therapy.

In March 2021, the patient returned to the gastroenterologist’s office again and the patient's LFTs relapsed after discontinuation of the prednisone; her ALT and AST were elevated at 55 and 163, respectively. Because of her relapse, she was started on 50 mg azathioprine once daily for long-term maintenance therapy. A seven-day prednisone treatment with a taper was also started, and repeat LFTs and a CBC were ordered. Later that month she had a lab follow-up with primary care; her ALT and AST were 59 and 79, respectively. She was not tolerating azathioprine due to side effects and was therefore started on 10 mg prednisone once daily due to elevated LFTs.

The patient did not follow up again until September 2021, now 59 years old. At this point, she was taking 5 mg prednisone daily for the management of AIH. Gastroenterology recommended that she taper off prednisone again and after which recheck her LFTs in an attempt to prevent long-term steroid use.

In October 2021, the patient had her annual wellness visit with primary care. LFTs were within normal limits. Her only complaints were dry eyes and a dry mouth. She discussed eye lubrication drops and false saliva and she preferred managing her symptoms with increased water intake and over-the-counter eye moisturizing drops. She continued maintenance of prednisone for AIH.

The patient followed up with rheumatology again in November 2021. She was started on 200 mg hydroxychloroquine twice daily and 2.5 mg of prednisone daily for SS and inflammatory arthritis.

**Figure 1:**
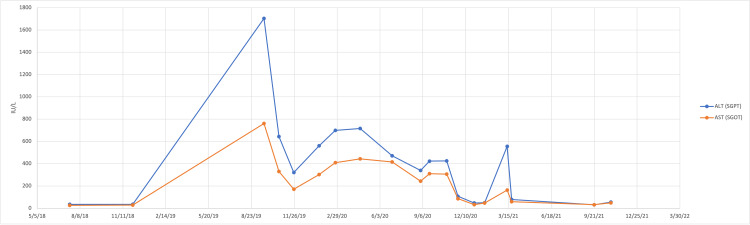
Trend of the patient’s ALT and AST over time. ALT: alanine aminotransferase; AST: aspartate aminotransferase; SGPT: serum glutamic pyruvic transaminase; SGOT: serum glutamic oxaloacetic transaminase.

In December 2021, the patient was seen for a routine primary care follow-up. Her ALT was mildly elevated at 41, and AST was within normal limits at 38. It was recommended that she continue hydroxychloroquine and prednisone as recommended by rheumatology. Figure 1 shows the changes in her ALT and AST over the time course in which she was being evaluated.

## Discussion

As displayed in Table 1, diagnosis of AIH depends on the fulfillment of four criteria: autoantibodies, hypergammaglobulinemia, liver histology, and absence of viral hepatitis [8]. In the aforementioned patient's case, labs including CMV, EBV, ANA with reflex, RF, ceruloplasmin, IgM, ESR, and CRP were obtained. Significant findings included a positive ANA and an anti-SSA > 8. Per liver biopsy performed in October 2020, no specific autoimmune etiology was identified. However, given the patient’s history of autoimmune disease and nonspecific biopsy, AIH including overlap with primary biliary cirrhosis, primary sclerosing cirrhosis, or drug-induced liver injury to infection could be compatible with the diagnosis. Negative viral hepatitis panels in both October 2019 and October 2020 confirm the absence of viral hepatitis. As IgG levels were not ordered, it is difficult to definitively classify the patient as meeting all four diagnostic criteria as mentioned in the 2008 simplified diagnostic criteria set forth by the International Autoimmune Hepatitis Group. Although compatibility with three of four diagnostic criteria was achieved in the patient’s workup, future concerns should emphasize obtaining all components displayed in Table 1 to offer a more accurate probability of AIH diagnosis.

Numerous studies have proposed a common underlying pathogenic mechanism present among systemic autoimmune disorders. Hepatology, in particular, demonstrates shared features between AIH, primary biliary cholangitis (PBC), primary sclerosing cholangitis (PSC), or indeterminate cholestasis. These so-called “overlap syndromes” can progress to cirrhosis or liver failure with inadequate treatment. In fact, AIH-PBC overlap syndrome ranges from frequencies of 10% to fewer than 2% of patients displaying PBC or AIH alone. Interestingly, as portrayed in the presented case, mutual immunological characteristics may also be shared between AIH-PBC overlap syndrome and SS [4].

Future studies should focus on unifying standards for autoantibody testing in diagnosing AIH [7]. In fact, previous data indicate that the simplified diagnostic criteria presented in Table 1 by the AIH group display a sensitivity of >80% and a specificity of >95% for AIH diagnosis [6]. However, standardization of diagnostic criteria between overlap syndromes continues to remain a challenge. Distinguishing between AIH and PBC and AIH and PSC was not always possible based on the use of a scoring system for simplified diagnostic criteria of AIH [7]. Nevertheless, clinical decision-making should act as the gold standard for overlapping syndrome diagnosis, with the strongest predictor in distinguishing between syndromes being a histopathologic examination of tissue from the liver [6]. Prompt AIH diagnosis can aid in the prevention of developing malignant disease; thus, it is imperative to make the diagnosis of cirrhosis in a timely fashion as several cases of hepatocellular carcinoma (HCC) in AIH cirrhosis have been reported [8]. Regular hepatic ultrasound screening for neoplastic foci may help in the detection and prevention of disease progression.

The prevalence of AIH in the presence of SS has not previously been well documented. The first association between primary Sjögren’s syndrome (pSS) and liver disease was reported in 1954 by Christiansson. Several case series in the late 1970s and early 1980s also recognized the likely association between liver disease and pSS. In fact, the incidence of an association between the two is perhaps more common when compared to liver disease and other autoimmune disorders; this may present valuable insight when navigating the aforementioned overlap syndromes [2].

A more aggressive workup for the patient in the case presentation began when LFTs showed abnormal values. Previous studies have demonstrated variations in the presence of AIH in patients with abnormal LFTs; however, a universal definition of “abnormal LFTs” is yet to be defined. It should be noted, however, that a majority of patients with any form of SS and abnormal LFTs have neither AIH nor PBC [2]. Therefore, the development of a protocol in patients presenting with abnormal LFTs in the presence of other autoimmune sequelae is needed.

## Conclusions

We reported a case of a woman who was diagnosed with AIH and SS after presenting to the clinic with elevated LFTs on routine blood work. While few studies highlight the relationship between AIH and SS, existing studies have shown that the prevalence of AIH is about 1% in patients with a diagnosis of pSS. Although the occurrence is rare, the diagnosis of both autoimmune diseases is important not only to guide therapeutic intervention but to also prevent long-term complications. Autoimmune diseases can present insidiously and share clinical features; thus, importance must be placed on thoroughly evaluating the patient and ordering the relevant lab work. In our case, IgG levels were not obtained for the patient, but are important for diagnostic criteria of AIH. Because the elevation in LFTs does not always correlate with the severity of liver damage, there is often a need for invasive testing, such as a liver biopsy, which some patients, as described in this report, may be hesitant to have done. Further studies should be aimed at understanding the relationship between concomitant autoimmune disorders so that treatment strategies can be optimized.
